# Correlations of magnetic resonance imaging classifications with preoperative functions among patients with refractory lateral epicondylitis

**DOI:** 10.1186/s12891-022-05651-9

**Published:** 2022-07-20

**Authors:** Xu Li, Yang Zhao, Zhijun Zhang, Tong Zheng, Shangzhe Li, Guang Yang, Yi Lu

**Affiliations:** Sports Medicine Service, Beijing Jishuitan Hospital, No. 31, Xin Jie Kou East Street, Xi Cheng District, Beijing, China

**Keywords:** Lateral epicondylitis, Magnetic resonance imaging, Lateral collateral ligament, Visual analog scale, Functional scores

## Abstract

**Background:**

To evaluate the correlations between three magnetic resonance imaging (MRI) classifications and preoperative function in patients with refractory lateral epicondylitis (LE).

**Methods:**

We retrospectively reviewed patients with refractory LE who underwent arthroscopic treatment. Signal changes in the origin of the extensor carpi radialis brevis (ERCB) were evaluated based on three different MRI classification systems. Spearman’s rank correlation analysis was used to analyse the correlation between each MRI classification and the preoperative functional and visual analogue scale (VAS). The lateral collateral ligament complex (LCL) in all patients was evaluated using both MRI and arthroscopy. The Mann–Whitney U test was used for the comparison of preoperative VAS and all functional scores between patients with refractory LE combined with LCL lesions, and those without.

**Results:**

There were 51 patients diagnosed with refractory LE between June 2014 to December 2020, all of whom were included in this study. The patients included 32 women and 19 men with a mean age of 49.1 ± 7.6 years (range, 39–60 years). The average duration of symptoms was 21.1 ± 21.2 months (range, 6–120 months). The intra-observer agreements for Steinborn et al.’s classification were 77.9%, 76.0%, and 76.7%, respectively. The inter-observer reliabilities of the three classifications were 0.734, 0.751, and 0.726, respectively. The average intra-observer agreement for the diagnosis of abnormal LCL signal was 89.9%, with an overall weighted kappa value of 0.904. The false-positive rate was 50%, and the false-negative rate was 48% for LCL evaluation on MRI. Spearman's rank correlation analysis did not find significant correlation between any of the three MRI classifications and preoperative VAS or any functional scores (all *P* > 0.05). There were no significant differences in the VAS and functional scores between patients with abnormal LCL signals on MRI and those without LCL lesions (all *P* > 0.05).

**Conclusions:**

Preoperative MRI findings in patients with refractory LE cannot reflect the severity of functional deficiency. Preoperative MRI grading of the origin of the ERCB and preoperative MRI for LCL signal change cannot assist the surgical plan for the treatment of patients with refractory LE.

## Background

Lateral epicondylitis(LE) of the elbow, also known as tennis elbow, is a tendinopathy/enthesopathy of the origin of the extensor carpi radialis brevis (ERCB) in the lateral epicondyle [1–3]. Previous histopathological studies have described indicators of LE which may include fibrovascular proliferation, intra-tendinous calcification, cartilage formation, fibrofatty degeneration, and partial or complete tendon ruptures [4–6].

Typically, the diagnosis of LE is made clinically by means of physical examination and examining the patient’s history, and does not necessitate advanced imaging modalities such as magnetic resonance imaging (MRI). Several conservative treatment methods have been proposed, including rest, physical therapy, massage, nonsteroidal anti-inflammatory drugs, and splinting/bracing; however, many patients still experience failed conservative treatment and develop refractory LE.

For refractory LE patients in whom conservative treatment regimens fail, MRI has been used to assess the severity of the origin of the ERCB lesions, excluding combined lesions, such as the lateral collateral ligament (LCL), to guide the surgical plan with higher sensitivity, specificity, and accuracy [7–11]. Alterations in signal intensity at the origin of the ERCB, particularly on T2-weighted sequences, have been described in previous studies with discrete fluid signals corresponding to focal lesions [6–21]. Based on these signal changes, different MRI classifications have been proposed, and results regarding the preoperative correlation between MRI classifications and clinical symptoms in refractory LE preoperatively are inconsistent [6, 7, 9, 11, 22]. Some authors have stated that a significant correlation exists between MRI findings and clinical symptoms [6, 7], however, other studies have failed to demonstrate a significant correlation between MRI findings and clinical symptoms [9, 11, 22].

MRI signal changes were also noted in the humeral attachment of the LCL due to its anatomical proximity to the original ERCB [23]. Despite the focus on ERCB signal changes and its relationship with patients’ clinical symptoms, no study has compared the functional difference in patients with refractory LE between those with combined LCL signal changes relative to those without LCL lesions using preoperative MRI images. The role of an MRI in the preoperative evaluation of patients with refractory LE remains controversial.

The aims of this study were to: (1) compare intra-reliability among three MRI classifications that are usually used in clinical practice; (2) evaluate the correlations among the three MRI classifications and clinical symptoms of refractory LE patients who need surgical intervention preoperatively; and (3) compare the preoperative function between refractory LE patients with an LCL signal change relative to those without LCL lesions.

## Methods

### Study design and participants

This retrospective study was approved by our Institutional Review Board. Informed consent was obtained from all the participants. The study involved reviewing and analyzing MRI images, intraoperative video and medical records of patients with a clinical diagnosis of refractory LE. All patients received conservative treatment for > 6 months, which included rest, physical therapy, massage, nonsteroidal anti-inflammatory drugs, and splinting/bracing; no invasive management was performed within 3 months before surgery. All patients underwent radiography of the elbow to exclude bony abnormalities.

### Inclusion criteria

We included patients: (1) aged between 18 and 60 years; (2) diagnosed with refractory LE; (3) without combined elbow posterolateral rotational instability; (4) normal range of motion of the involved elbow joint; and (5) with complete preoperative medical records and willing to undergo an intraoperative arthroscopic examination.

### Exclusion criteria

We excluded those with: (1) Age < 18 years or > 60 years; (2) glucocorticoid- or platelet- rich plasma injections within the past 3 months before surgical treatment; (3) inflammatory diseases or osteoarthritis of the elbow joint; (4) diabetes mellitus; (5) neuropathy of the brachial plexus; (6) elbow instability; and (7) limited range of motion compared to the healthy contralateral side.

Additional exclusion criteria were: (1) prior upper extremity injury or surgery; (2) bilateral LE or LE combined with medial epicondylitis; and (3) comorbidities that could interfere with the ability to participate in this study.

### Preoperational functional assessments

The primary outcome measure was the Disabilities of the Arm, Shoulder and Hand (DASH) outcome measure [24]. The DASH questionnaire is a region-specific questionnaire that has shown reliability, validity, and responsiveness in both proximal and distal disorders of the upper extremities. The Mayo Elbow Performance Score (MEPS) [25], which is the sum of the pain, stability and function subscales, ranges from 0 to 100, with 0 being the best score and 100 being the worst score. Patient-reported function was evaluated using the Patient-Rated Tennis Elbow Evaluation (PRTEE) [19], a validated disease-specific measure composed of five questions on pain and 10 on function, using a series of 10-point Likert scales. A 0–10 visual analog scale (VAS) was used to assess pain intensity at rest and during daily life activities, with 0 indicating no pain and 10 indicating the maximum possible pain.

### MRI Evaluation

All MRI evaluations were performed using a 1.5-Tesla MR system (Philips Healthcare, Best, Netherlands). The patients were placed in the supine position with the arm along the side of the body, elbow extended, and wrist supinated. Coronal proton density (PD) turbo spin echo (TSE) spectral attenuated inversion recovery (SPAIR) (TR = 2500 ms, TE = 36 ms), coronal T1-weighted TSE (TR = 700 ms, TE = 8 ms), sagittal PD TSE SPAIR (TR = 2600 ms, TE = 36 ms) and axial PD TSE SPAIR (TR = 2600 ms, TE = 37 ms) were obtained, with 2-mm slices and a 120–130 mm field of view. All MRI evaluations were performed within 1 week before surgery.

**Fig. 1:**
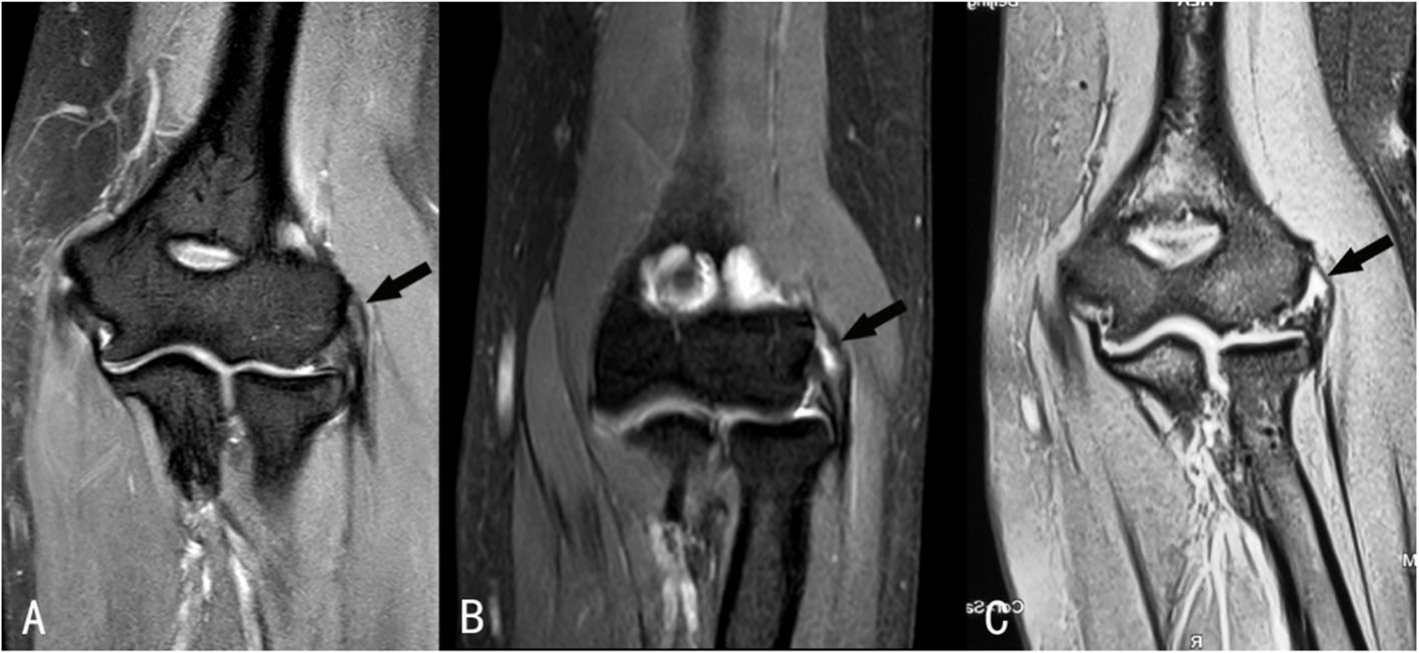
Examples of MRI findings on the origin of the ERCB, the grades are same within the three classifications. **A** 44-year-old male, MRI showed grade 1 injury on the origin of the ERCB (focal or mild increased signal intensity). **B** 48-year-old female, MRI detected grade 2 injury on the origin of the ERCB (Moderate increased signal intensity, comprise less than 50% of the tendon thickness). **C** 52-year-old female, MRI detected grade 3 injury on the origin of the ERCB (Severe increased signal intensity, comprise more than 80% of the tendon thickness) (black arrow)

The pathologies of the origin of ERCB in all patients with refractory LE were classified separately based on three different MRI classifications (Fig. 1). The three classifications are as follows:

Modified Steinborn classification [10]

- 0- Dark tendon, no signal intensity changes
- 1- Focal area of increased signal intensity without tendon thickening
- 2- Area of increased signal intensity involving < 50% of tendon cross section with tendon thickening
- 3- Area of increased signal intensity involving > 50% of tendon cross section with tendon thickening

Rabago classification [18]

- 0- Normal tendon with uniform low signal intensity
- 1- Mild tendinopathy which is thickened and has intermediate signal intensity
- 2- Moderate tendinopathy which is thinned and shows focal areas of intense fluid-like signal intensity which comprise less than 50% of the tendon thickness
- 3- Severe tendinopathy which is thinned and shows focal areas of intense fluid-like signal intensity which comprise more than 50% of the tendon thickness

Walz classification [21]

- 0- Complete homogenous low intensity
- 1- Mild epicondylitis is characterized by tendon thickening and increased internal signal intensity, affecting less than 20% of the tendon thickness
- 2- Partial-thickness tear with thinning and focal disruption that does not extend across the full thickness of the tendon, affecting between 20% to 80% of the tendon thickness
- 3- Complete or complete tear, characterized as a fluid-filled gap separating the tendon from its origin at the lateral epicondyle, affecting more than 80% of the tendon thickness

**Fig. 2:**
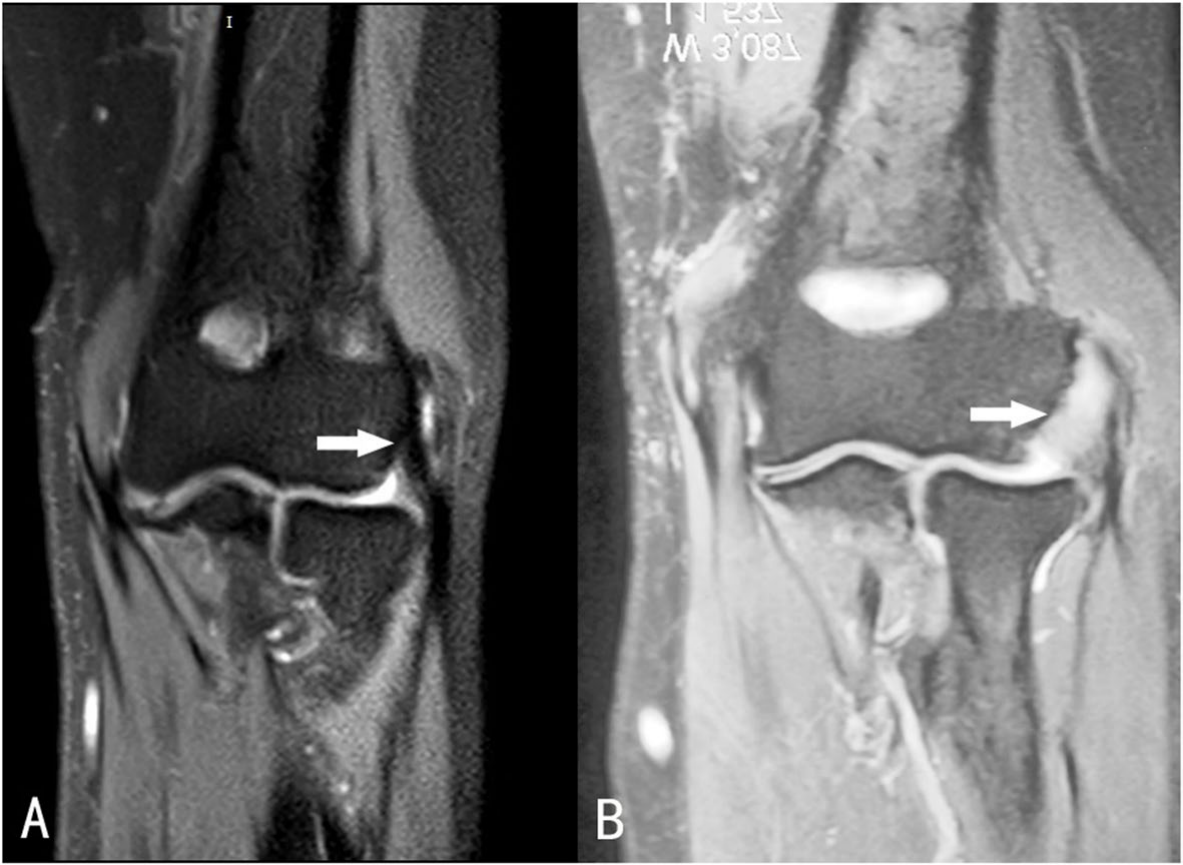
Examples of MRI findings on the LCL. **A** 53-year-old female, MRI showed normal low intensity signal of the LCL. **B** 49-year-old male, MRI detected abnormal higher intensity signal of the LCL (white arrow)

The LCL of all patients was also assessed by MRI and classified as normal if completely homogenous at low intensity, and abnormal if the signal had to be intensified in the ligament area (Fig. 2).

Preoperative MRI scans of all patients’ were assessed independently by three musculoskeletal radiologists who were blinded to the patients’ preoperative functional information and surgical records. Each radiologist graded the MRI of all patients on two separate occasions, with a one month delay between each rating

### Arthroscopic evaluation

Each patient’s surgical video record was reviewed and evaluated by two independent surgeons. Any discrepancy was resolved by an external senior surgeon for the origin of both the ERCB and LCL lesions. The origin of the ERCB was evaluated through the proximal anteromedial portal after the anterior capsule release. The LCL was evaluated through the soft spot portal after radio-humeral joint debridement.

### Statistical analysis

All analyses were conducted using the SPSS software (version 13.0; Chicago, Illinois, U.S.). Analyses were performed for each classification of the MRI scoring system and the preoperative functional evaluation score. The Fleiss’ kappa statistic was used to determine the inter- and intra-observer reliability of each MRI classification. kappa values from 0.41 to 0.60 were considered fair, 0.61 to 0.80 good, and > 0.81 excellent. Spearman’s rank correlation analysis was used to analyse the correlations between each MRI classification and the preoperative VAS, MEPS, DASH, and PRTEE scores. The Mann–Whitney U test was used to compare preoperative VAS, MEPS, DASH, and PRTEE scores between patients with lesions and those without LCL lesions. Statistical significance was set at *P* < 0.05.

## Results

There were 51 patients with a clinical diagnosis of refractory LE, all of whom had operations occurring from June 2014 to December 2020. All the patients were treated arthroscopically. There were 32 women and 19 men with a mean age of 49.1 ± 7.6 years (range, 39–60 years). The average duration of symptoms was 21.1 ± 21.2 months (range, 6–120 months). The left side/right side ratio was 16:35, dominant side/non-dominant side ratio was 42:9, and heavy work/light work ratio was 28:23.

The intra-observer agreements for Steinborn et al.’s classification were 77.9%, 76.0%, and 76.7%, respectively. The inter-observer reliabilities of the three classifications were 0.734, 0.751, and 0.726, respectively, indicating that all three classifications demonstrated good reliability (Table 1). The average intra-observer agreement for the diagnosis of abnormal LCL signal was 89.9%, with an overall weighted kappa value of 0.904, which indicates excellent inter-observer reliability.

**Table 1:** Distribution of three types of MRI classification and intra-reliability

Grades	0	1	2	3	Intra-reliability
Steinborn	0	14	10	27	0.734
Rabago	0	13	11	27	0.751
Walz	0	13	9	29	0.726

The preoperative VAS, MEPS, DASH, and PRTEE scores were available for 51 patients. There was no significant positive correlation by Spearman’s rank correlation analysis between degrees of MRI scores and VAS scores or any functional evaluation scores (Table 2).

**Table 2:** Correlations of three MRI classifications and preoperational functional scores

Classification		RVAS	MVAS	MEPS	DASH	PRTEE
Steinborn	*R* value	0.08	0.174	-0.042	0.036	0.079
	*P* value	0.57	0.212	0.767	0.798	0.573
Rabago	*R* value	0.02	0.221	-0.097	0.092	0.049
	*P* value	0.885	0.113	0.49	0.511	0.727
Walz	*R* value	0.104	0.196	-0.084	0.072	0.115
	*P* value	0.460	0.159	0.549	0.611	0.414

Values are expressed as mean ± SD, *RVAS* Visual Analog Scale at Rest, *MVAS* Visual Analog Scale during daily life activity, *MEPS* Mayo Elbow Performance Score, *DASH* Disabilities of the Arm, Shoulder and Hand, *PRTEE* Patient-Rated Tennis Elbow Evaluation

Overall, 28 patients showed abnormal LCL signals, while 23 patients had a normal appearance of the LCL on their preoperative MRI scans. Among the 23 patients with normal LCL on MRI, 12 were confirmed to have degenerative ligament pathology by viewing surgical records. Among the 28 patients with abnormal LCL signal changes, 13 exhibited normal LCL appearance based on surgical records. The false-positive rate was 50%, and the false-negative rate was 48%, which was relatively high. All the LCL lesions showed ligament abrasion or degeneration. No partial or complete tears of the LCL were observed. There were no significant differences in VAS and all functional evaluation scores between patients with refractory LE and those with normal or abnormal LCL signals on MRI (Table 3).

**Table 3:** Comparison between groups based on LCL features on MRI

Functional Scores	Abnormal LCL (*N* = 28)	Normal LCL (*N* = 23)	*P* value
RVAS	1.9 ± 2.5	1.7 ± 2.6	0.515
MVAS	5.4 ± 2.1	4.8 ± 2.1	0.162
MEPS	68.0 ± 14.3	71.3 ± 11.1	0.490
DASH	44.4 ± 8.5	44.4 ± 6.9	0.964
PRTEE	51.4 ± 19.3	51.5 ± 18.3	0.894

Values are expressed as mean ± SD, *LCL* Lateral Collateral Ligament, *RVAS* Visual Analog Scale at Rest, *MVAS* Visual Analog Scale during daily life activity, *MEPS* Mayo Elbow Performance Score, *DASH* Disabilities of the Arm, Shoulder and Hand, *PRTEE* Patient-Rated Tennis Elbow Evaluation

## Discussion

LE is a clinically diagnosed disease. However, the use of MRI remains controversial. The most important finding of the current study was that there were no significant correlations between the three MRI classifications and the VAS and functional evaluation scores preoperatively, which indicates that the pathologic appearance of the origin of the ERCB had no correlation with preoperative functional deficiency among refractory LE patients. In addition, there were no significant differences on refractory LE preoperative function between the patients with refractory LE and those with concomitant LCL lesions.

Abnormal features of the origin of the ERCB can be found on MRI in patients with LE. A normal ERCB origin usually shows a homogenous low signal on both T1-weighted and T2-weighted MRI images. When tendinopathy/enthesopathy of the origin of the ERCB occurred, an increased signal intensity was observed on MRI. Martin reported that all their twenty-four epicondylitis patients had an increased signal on fat-saturated FSE and fast STIR images [8]. Mackay et al. [15] found signs of oedema around the ERCB origin in all twenty-three symptomatic tennis elbows and six out of 17 asymptomatic elbows. In the present study, abnormal signals of the origin of the ERCB on MRI existed in all refractory LE patients, who were confirmed arthroscopically; these findings are comparable with results from previous studies [9–11, 16]. In the present study, three different MRI classifications that are typically used in clinical practice were examined in our analysis. Good intra- and inter-observer reliabilities for the evaluation of ERCB on MRI were observed in the current study. The intra-observer agreement for the three classifications was 77.9%, 76.0%, and 76.7%, and the inter-observer reliabilities of the three classifications were 0.734, 0.751, and 0.726, respectively. The current study is comparable with previous studies [7, 17] and proves again that MRI differentiates the degree of ECRB lesions in patients with refractory LE.

Although the excellent and good reliability of MRI has been proven to show pathologic changes in the origin of the ERCB in LE patients [7], correlations of findings of the origin of the ERCB with clinical function of refractory LE have not been well established. Potter et al. [16] considered the use of MRI in recalcitrant LE assists in surgical planning because MRI findings in 21 patients correlated with the surgical findings of primary degeneration of ECRB. Qi et al. [17] reviewed tendinopathy in 96 LE patients on MRI images and found that the severity of MR signal changes of the origin of the ERCB positively correlated with the PRTEE scores On the other hand, Walton et al. [11] stated in his study that there was no correlation between MRI results and clinical symptoms. Chourasia et al. [22] found no statistically significant association between MRI findings and PRTEE. Savnik et al. [9] ound no significant difference in VAS scores between patients with or without ERCB signal changes on MRI and concluded that MRI images did not imply the need for surgery. Based on this discrepancy, the necessity of preoperative MRI should be determined as a clinical protocol.

The present study did not find a significant correlation between each classification and any preoperative function evaluation system, which may imply that the degree of signal change of the origin of the ERCB found on MRI is, may not serve as a strong indicator of preoperative functional deficiency among refractory LE patients.

Steinborn and colleagues [10] identified T1 signal changes at the origin of the ERCB in 6 and T2 signal changes, suggesting a defect in 3 of 11 asymptomatic elbows. van Kollenburg et al. [20] found 19% MRI signal abnormalities in their control group compared to those in the LE group. van Leeuwen et al*.* [26] identified tendon signal changes on MRI in 369 of 3374 patients (11%) without a clinical suspicion of LE, and the prevalence of incidental signal changes in the origin of the ERCB increased gradually with age regardless of symptoms. All of these previous studies doubt the merit of the clinical use of MRI in patients with LE. Although all three MRI classification systems can differentiate the degree of the origin of the ERCB lesions with high reliability in the present study, there was no relationship between different degrees of ECRB lesions and preoperational functional deficiency. These findings should signal that caution should be exercised when implementing MRI scans in the preoperative period among patients diagnosed with refractory LE.

From previous studies, LCL complex abnormality was reported as the most common accompanying finding in LE patients and can be considered a risk factor for failure of conservative therapy [7, 14]. However, no complete or partial tear of the LCL was confirmed arthroscopically in the current study, and no significant differences were found in any preoperative functional evaluation system between patients with concomitant LCL signal changes and those without. There were no instability complaints with negative medial and lateral stability tests in the series, which means that the lesion of the LCL was still in the early stages, and the consistency of the ligament was maintained in all patients in the present study. This may explain the discrepancy with the results of other studies.

Meanwhile, the accuracy of the MRI images was low, the false-positive rate of the LCL injury was 50%, and the false-negative rate was 48% after arthroscopic confirmation. van Kollenburg et al. [20] also proved that the incidental abnormal finding in the LCL on MRI was common in control patients with other elbow issues. Overinterpretation of the signal abnormalities in the LCL associated with LE may lead surgeons to consider a more invasive surgical procedure that is not merited.

The results of the current study imply that although preoperative MRI in patients with LE may detect signal changes in the origin of the ERCB and LCL of the elbow joint, it cannot reflect the severity of the patients’ symptoms. Preoperative MRI grading of the origin of the ERCB and preoperative MRI for LCL signal change cannot assist the surgical plan for the treatment of patients with refractory LE.

The present study has some limitations. First, it featured a retrospective design and a relatively small sample size, and clinical variables were collected from medical records, which may have led to analysis bias. Second, MRI images of the healthy side of the patients were not evaluated, as a healthy population may exhibit abnormal signals on the origin of the ERCB. However, all origins of the ERCB and LCL lesions were confirmed arthroscopically, which may exclude the possibility of a false positive. Third, the MRI grading or patients’ VAS and functional scores were not analyzed with arthroscopic findings or postoperative outcomes, as this study focused mainly on the relationship between the MRI findings of the origin of the ERCB and patients’ preoperative VAS and functional scores. Finally, grip strength was not evaluated preoperatively, which may reflect the differences between the origins of the ERCB signal changes. Since most patients were unwilling to perform a power test due to their experienced pain, more functional scores to address elbow function were evaluated in the current study.

## Conclusion

Preoperative MRI in patients with refractory LE cannot reflect the severity of the functional deficiency in these patients. Preoperative MRI grading of the origin of the ERCB and preoperative MRI for LCL signal change should not be used to assist the physician’s surgical plan when treating patients with refractory LE. Other parameters should be explored to predict the postoperative outcomes in patients with refractory LE.

## Data Availability

The data sets used and/or analyzed during the current study are available from the corresponding author on reasonable request.

## Abbreviations

MRI: Magnetic resonance imaging
LE: Lateral epicondylitis
ERCB: Extensor carpi radialis brevis
VAS: Visual analog scale
LCL: Lateral collateral ligament
DASH: Disabilities of the arm, shoulder and hand
MEPS: Mayo elbow performance score
PRTEE: Patient-rated tennis elbow evaluation
PD: Proton density
TSE: Turbo spin echo
SPAIR: Spectral attenuated inversion recovery
